# Vitamin C and D Deficiency in Urban America: A Case Report

**DOI:** 10.5811/cpcem.1253

**Published:** 2023-12-20

**Authors:** Alyssa A. Lombardi, Maura Sammon, Kraftin E. Schreyer

**Affiliations:** Temple University Hospital, Department of Emergency Medicine, Philadelphia, Pennsylvania

**Keywords:** *scurvy*, *ascorbic acid*, *vitamin D*, *malnutrition*, *case report*

## Abstract

**Introduction:**

Scurvy is caused by vitamin C deficiency and manifests with a variety of symptoms including generalized fatigue, apathy, anemia, myalgias, easy bruising, and poor wound healing. It is generally thought of as a disease of the past, especially in developed countries. However, vitamin C deficiency still occurs, especially in patients with lack of access to fruits and vegetables. Other micronutrient deficiencies, including vitamin D deficiency, are also prevalent and can cause a multitude of signs and symptoms including osteomalacia, muscle weakness, and increased risk of many chronic illnesses.

**Case Report:**

Here we present a case of vitamin C and D deficiency in a previously healthy 26-year-old man during the coronavirus disease 2019 pandemic in urban America.

**Conclusion:**

Severe nutritional deficiencies still exist today. Emergency clinicians should be aware of the signs and symptoms to promptly diagnose and initiate treatment.

## INTRODUCTION

Vitamin C (ascorbic acid) is a vital piece of many biosynthetic pathways. However, humans are unable to synthesize ascorbic acid and must obtain it from their diet.^1^ Clinically, it is well documented that vitamin C is crucial to prevent scurvy, which presents with fatigue, malaise, anemia, petechia, perifollicular hemorrhages, poor wound healing, and depression.^1^ ^,^ ^2^ Scurvy is often thought of as an ancient disease that afflicted sailors deprived of fruits and vegetables.^3^ We describe a case of a 26-year-old man who presented with scurvy during the coronavirus disease 2019 (COVID-19) pandemic. This case shows the importance of keeping nutritional deficiencies on the differential, especially with risk factors such as isolation, food insecurity, old age, restrictive diets, malabsorption syndromes, or substance use disorder.

## CASE REPORT

A 26-year-old male with a past medical history of depression presented to our emergency department (ED) twice, first in February 2021 and again in March 2021. On his initial visit, his chief concern was leg pain. The patient reported that his symptoms began as bilateral foot and low back pain about three months prior to arrival. While the back pain resolved, his foot pain progressed up his bilateral lower extremities, which had become stiff. Over the prior several weeks, he had begun to have difficulty extending his knees. He additionally noted easy bruising and small red dots all over his extremities. He denied fever, vomiting, diarrhea, abnormal bleeding, weight loss, and prior spinal surgeries. There was no known family history of rheumatologic, immunologic, metabolic, or connective tissue disorders. The patient did not drink alcohol, smoke, or use illicit substances. He did note that he had been eating pizza bagels since April 2020. He drank only water. He did not take any vitamin supplementation. At the time he was not employed and lived with a roommate. He had not been evaluated previously for these concerns.

The patient was afebrile, with a temperature of 98.1° Fahrenheit (F). He had a heart rate of 91 beats per minute (bpm), blood pressure 128/89 millimeters of mercury (mm Hg), and a respiratory rate of 16 breaths per minute. His oxygen saturation was 99% on room air. He weighed 61.2 kg. On physical examination, he was pale and thin. He had contractures in the bilateral lower extremities with healing ecchymoses in the flexor surfaces of bilateral thighs and knees. Perifollicular hemorrhages were noted diffusely in the bilateral upper and lower extremities (Image). Petechiae were noted on the bilateral lower extremities. He had full strength in the bilateral upper and lower extremities, and intact sensation in all extremities. He had a normal thyroid exam. There was no gingival bleeding. He had a negative Steinberg sign, also known as the thumb sign. This test is considered positive if the distal phalanx of the adducted thumb extends beyond the ulnar border of the palm when clenching one’s fist (suggestive of Marfan disease).

**Image:**
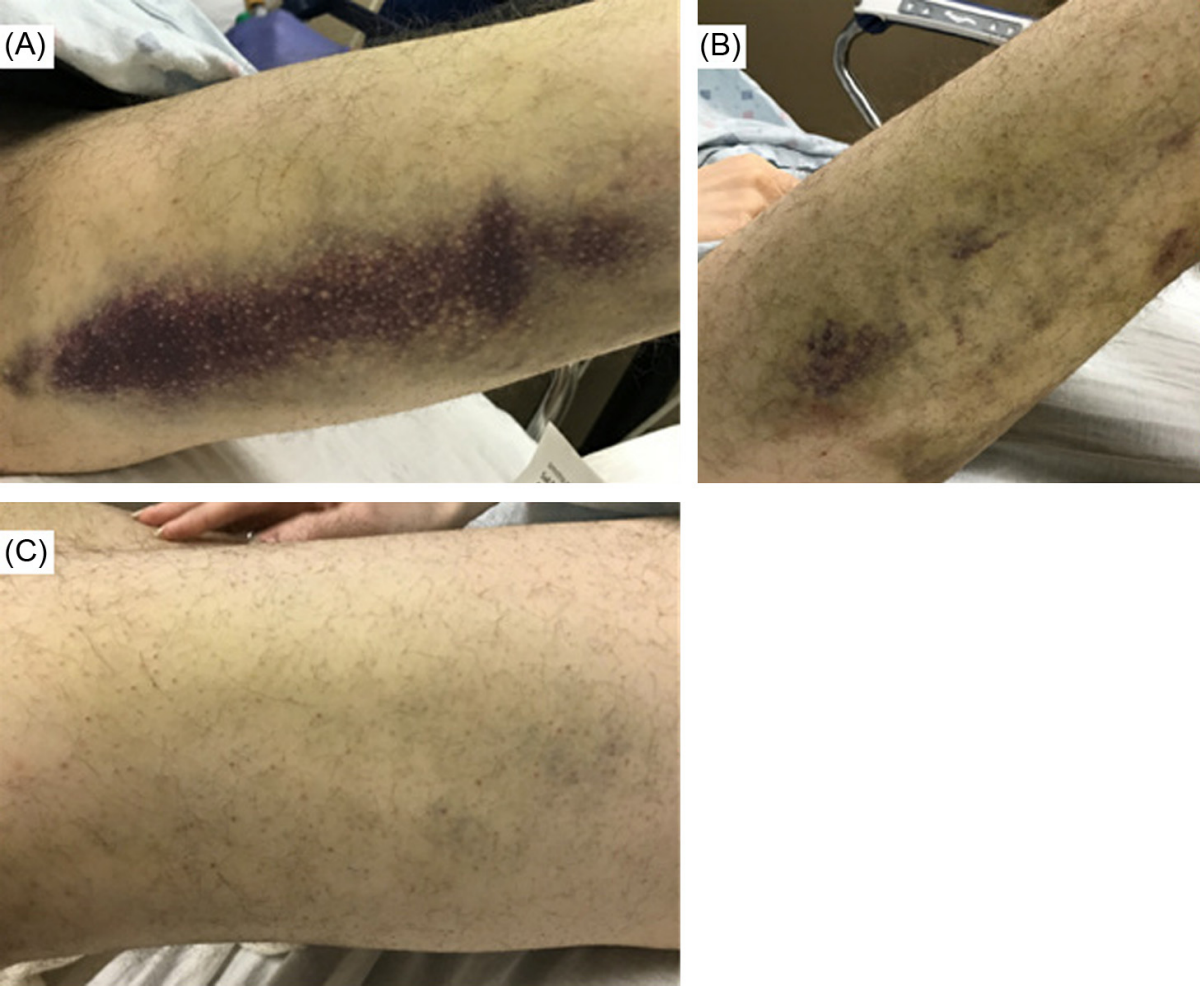
(A) Spontaneous ecchymoses on patient’s left thigh. (B) and (C) Perifollicular hemorrhages and petechiae of the patient’s right (B) and left (C) lower extremities.

His initial laboratory evaluation demonstrated a hemoglobin of 11.0 grams per deciliter (g/dL) with a mean corpuscular volume of 88.7 femtoliters (fL), a white blood cell count of 7.7 × 10^3^/milliliters (K/mL), and a platelet count of 321 K/mm^3^. The creatinine was 0.69 milligrams (mg)/dL), potassium 3.9 millimoles per liter, magnesium 2.3 mg/dL, calcium 8.4 mg/dL, and phosphorus 2.9 mg/dL. The albumin was 3.4 g/dL, and indirect bilirubin was elevated at 1.3 mg/dL (Table). He was discharged with hematology follow-up given the anemia and bruising.

**Table.** Summary of scurvy patient’s lab results during his two emergency department visits.

Lab	Unit	Reference range	1^st^ ED Visit February 2021	2^nd^ ED Visit March 2021
Hemoglobin	g/dL	14–17.5	11.0	8.5
Mean corpuscular volume	fL	80.00–96.0	88.7	87.9
White blood cells	K/mL	4.0–11.0	7.7	3.6
Platelets	K/mL	150–450	321	306
Creatinine	mg/dL	0.80–1.40	0.69	0.66
Potassium	mmol/L	3.5–5.2	3.9	3.8
Magnesium	mg/dL	1.7–3.0	2.3	2.1
Calcium	mg/dL	8.6–10.0	8.4	7.8
Phosphorus	mg/dL	2.8–4.5	2.9	3.7
Albumin	g/dL	3.5–5.0	3.4	3.4
Total bilirubin	mg/dL	0.0–1.0	1.3	1.3

*g/dL*, gram per deciliter; *fL*, femtoliters; *K/mm* ^3^, thousand cells per cubic millimeter; *mg/dL*, milligram per deciliter; *mmol/L*, millimole per liter; *ED*, emergency department.

He presented to the hematology clinic in March 2021 and was immediately referred back to the ED. At that time, he was unable to ambulate and instead was moving around by pulling himself with his arms. On the second visit, he had a temperature of 97.3°F, heart rate of 94 bpm, blood pressure 94/57 mm Hg, and respiratory rate 20 breaths per minute. His oxygen saturation was 99% on room air. He weighed 59.9 kg. His physical examination was notable for progressive contractures in his bilateral lower extremities. He had pain and tenderness at tendon and ligament insertions, suggesting enthesitis and preventing knee extension. Additionally, he had contracture of the right elbow. Ecchymoses were also visible on the right upper extremity. He was noted to be apathetic and indifferent to his current physical condition. His repeat laboratory evaluation is noted in the table.

Diagnostic considerations included nutritional disorders, specifically, scurvy and Vitamin D deficiency, Ehlers-Danlos syndrome, scleroderma, coagulopathy secondary to factor XIII deficiency, alpha 2-antiplasmin, or plasminogen activator inhibitor-1 deficiency. Scurvy was highest on our differential based on his constellation of signs and symptoms. He was admitted for further evaluation and care.

During his hospital stay, he was treated presumptively for scurvy with ascorbic acid. The vitamin C level later resulted at 0.1 mg/dL (reference range: 0.4–1.7 mg/dL). He was also treated for vitamin D deficiency after his vitamin D level resulted at 9.7 nanograms per milliliter (ng/m) (30–50 ng/mL). He was evaluated by nutrition, physical therapy, physical medicine and rehabilitation, dermatology, and endocrinology. He was ultimately discharged to a skilled nursing facility on ascorbic acid and ergocalciferol.

He completed treatment with ascorbic acid and high-dose ergocalciferol after 12 weeks. His symptoms, contractures, and skin findings resolved, and he was able to resume activities of daily living, including ambulation. He gained 5.4 kg. He continues to follow up with internal medicine and psychiatry. The figure above outlines the timeline of this case.

**Figure:**
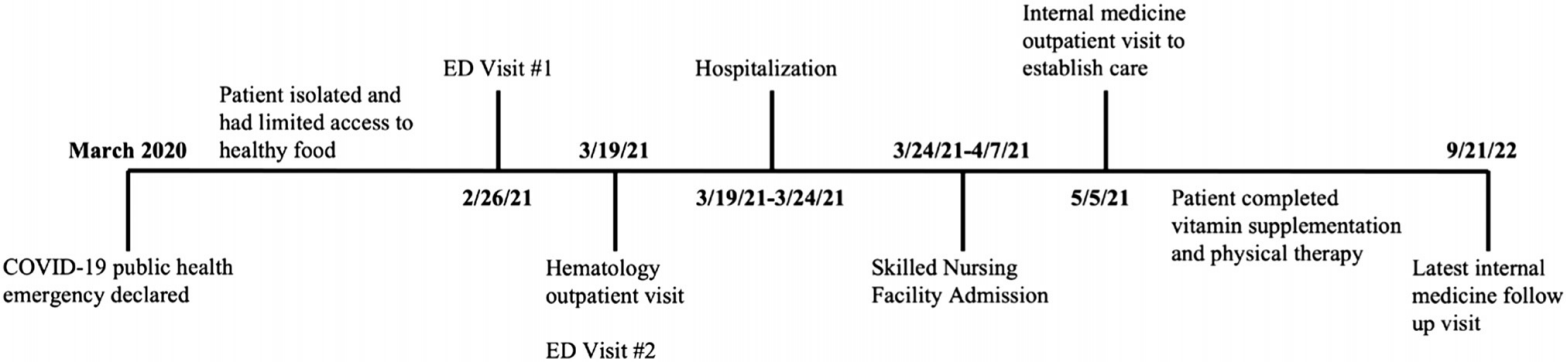
Care timeline of a patient diagnosed with scurvy. *COVID-19*, coronavirus disease 2019; *ED*, emergency department.

## DISCUSSION

Scurvy is caused by vitamin C deficiency and manifests with multisystem organ involvement.^1^ ^,^ ^3^ The relationship between scurvy and citrus fruits was first described by James Lind in 1753, and subsequent research showed that the reason citrus fruits prevent scurvy is because they contain ascorbic acid.^1^ ^,^ ^2^ Humans are not able to derive ascorbic acid from glucose metabolism (unlike other animals); so, they need regular dietary intake of vitamin C.^1^ In addition to citrus fruits, vitamin C is found in potatoes, tomatoes, berries, and green vegetables. It is important to note that cooking these items reduces their vitamin C content.^4^ This is probably why our patient developed scurvy despite eating tomatoes.

Ascorbic acid is involved in several important biosynthetic pathways including collagen assembly, amino acid metabolism, synthesis of norepinephrine, and iron absorption.^1^ Accordingly, the clinical presentation of scurvy manifests with multiorgan system involvement reflective of the multitude of functions vitamin C plays in the body. After about 60–90 days without sufficient vitamin C, scurvy develops.^5^ Constitutional symptoms often present first with significant fatigue, progressive weakness, myalgias, and lassitude. Physical exam often displays specific cutaneous signs including follicular hyperkeratosis associated with coiled corkscrew hairs, perifollicular hemorrhages, petechiae, purpura, and ecchymoses. Other manifestations include gingival bleeding, joint pain, hemarthrosis, and anemia.^1^ ^,^ ^5^ Scurvy is curable with vitamin C repletion.^2^ ^,^ ^5^

In addition to scurvy, our patient also had severe vitamin D deficiency. Humans need regular dietary intake of vitamin D and/or sunlight exposure. Foods naturally containing vitamin D include fish, eggs, mushrooms, and liver. In the United States, vitamin D is often added to milk. Vitamin D deficiency is most well known for causing rickets in children and osteomalacia in adults. Vitamin D deficiency has also been associated with certain infections, autoimmune diseases, dementia, and depression.^6^ ^–^ ^8^

In industrialized countries, severe nutritional deficiencies are uncommon; however, many people have inadequate intake of micronutrients. Data from the National Health and Nutrition Examination Survey from 2005–2016 revealed that the prevalence of inadequate vitamin C intake was 46% and the prevalence of inadequate vitamin D intake was 95%.^7^

In this case, the COVID-19 pandemic led to unemployment and social isolation. As a result, the patient was only eating pizza bagels (which he made with bagels, tomato sauce, cheese, and pepperoni) and drinking water, which led to severe deficiencies of vitamins C and D. This case illustrates the importance of keeping nutritional deficiencies on the differential, especially when there are risk factors such as isolation and food insecurity.

## CONCLUSION

Severe micronutrient deficiencies have significant clinical consequences. More specifically, vitamin C is involved in several important biosynthetic pathways, and deficiency results in a constellation of signs and symptoms that includes perifollicular hemorrhage, corkscrew hairs, purpura, gingivitis, arthralgias, poor wound healing, and fatigue. Having a high clinical suspicion for nutritional deficiencies is key to diagnosis and treatment.
